# A Noninvasive Retrograde Flushing System for Shunted Hydrocephalus: Initial Case Series of 25 Patients

**DOI:** 10.7759/cureus.8940

**Published:** 2020-07-01

**Authors:** Joseph Falcone, Cindy Ho, Ramin Eskandari, Sudhakar Vadivelu, Joseph R Madsen, Michael G Muhonen

**Affiliations:** 1 Neurosurgery, University of California, Irvine, USA; 2 Neurosurgery, Children's Hospital of Orange County, Orange County, USA; 3 Neurosurgery, Medical University of South Carolina, Charleston, USA; 4 Neurosurgery, Cincinnati Children's Hospital Medical Center, Cincinnati, USA; 5 Neurosurgery, Boston Children's Hospital/Harvard Medical School, Boston, USA

**Keywords:** hydrocephalus, cerebrospinal fluid shunt, shunt failure, shunt obstruction

## Abstract

Hydrocephalus is a common neurosurgical pathology associated with high patient morbidity and systemwide healthcare costs. A significant portion of these costs are related to the failure of ventricular shunting systems. Despite decades of research and technological development, the rate of shunt failure and revision has not significantly improved. The Reflow™ Ventricular System (Anuncia, Inc., Lowell, MA) is a recent technological development with the potential to prolong the shunt lifespan. This system introduces a noninvasive means of flushing a shunt proximally with a controlled, repeatable pulse of cerebral spinal fluid (CSF) and of creating a new ventricular opening for occluded shunts. In this multicenter case series, we present the early clinical experiences with this device and discuss its potential.

## Introduction

Hydrocephalus is a common neurosurgical pathology, with a disproportionate degree of associated morbidity and resource utilization. Nearly 400,000 new cases of pediatric hydrocephalus are estimated annually worldwide [1], and adult-onset hydrocephalus is a growing phenomenon [2] partly related to increased recognition of normal pressure hydrocephalus [3].

These patients impose a heavy burden on healthcare systems. United States (US) hospitals discharge approximately 69,000 patients annually with a primary diagnosis of hydrocephalus, of which half undergo placement of a new shunt [4]. A review of the Nationwide Inpatient Sample database for 2000 in the US saw 27,870 patients undergo shunt-related procedures, 42.8% of which were replacements of existing shunts [5]. Over half of these admissions were classified as emergent or urgent, and the average cost was found to be 35,816 US dollars each [5].

These costs represent a significant economic burden, which is disproportionate to the disease incidence. A cross-sectional review of Healthcare Cost and Utilization Project Kids' Inpatient Databases showed that in 2003, hydrocephalus accounted for 0.6% of all pediatric hospital admissions but 3.1% of all pediatric hospital charges [6]. Shunting procedures are estimated to account for over 100 million US dollars of national health care expenditures annually, half of which is related to shunt revision [7]. Because of this, significant research emphasis has been placed on reducing these costs, with cost analyses identifying shunt failure as the factor with the greatest potential system-wide cost savings [8].

One recent technological development that has the potential for an impact on these economic and patient outcome statistics is the Reflow™ Ventricular System (Anuncia, Inc., Lowell, MA, USA). This system introduces two novel functions to a ventricular shunt, allowing controlled retrograde flushing into the proximal catheter, as well as a “relief membrane” that can create a new intraventricular opening in a shunt with obstructed inlet holes. In this paper, we report the first experiences with this device and discuss preliminary evidence of its potential utility.

## Materials and methods

This is a multicenter case series review of the first 25 consecutive patients in which the Reflow™ system has been employed at four US pediatric hospitals. Twenty-five patients were implanted with the Reflow™ device between May 2018 and November 2019. Operations were performed at Children’s Hospital of Orange County (CHOC), Boston Children’s Hospital (BCH), Medical University of South Carolina (MUSC), and Cincinnati Children’s Hospital and Medical (CCHM).

Inclusion criteria involved a diagnosis of shunt-dependent hydrocephalus in both pediatric and adult patients with either a history of frequent shunt failure or clinical factors indicating a high risk of shunt obstruction, including recent infection, hemorrhage, high cerebrospinal fluid (CSF) protein counts, or chronically collapsed ventricles. Patients with active infection underwent appropriate antibiotic management with external ventricular drainage prior to the placement of the VP shunt. Infants were excluded from this study due to the possibility of skin erosion in this population given the prominent profile of the device.

Baseline patient characteristics were collected, including age, sex, and etiology of hydrocephalus. Information such as preoperative CSF profile and protein count, as well as radiographic characterization of ventricular systems was not uniformly available among the participating sites. Outcomes were assessed based on clinical data including presentation with concern for shunt failure, use of the flushing system, as well as any complications that may be attributed to the device.

The Reflow™ system

The Reflow™ system introduces two new components to a traditional shunt system: a retrograde flushing device and a “relief-membrane” on the proximal catheter that can open in the setting of occlusion. This system has undergone rigorous preclinical testing and received US Food and Drug Administration (FDA) clearance. It received CE marking for the treatment of hydrocephalus in 2017.

**Figure 1:**
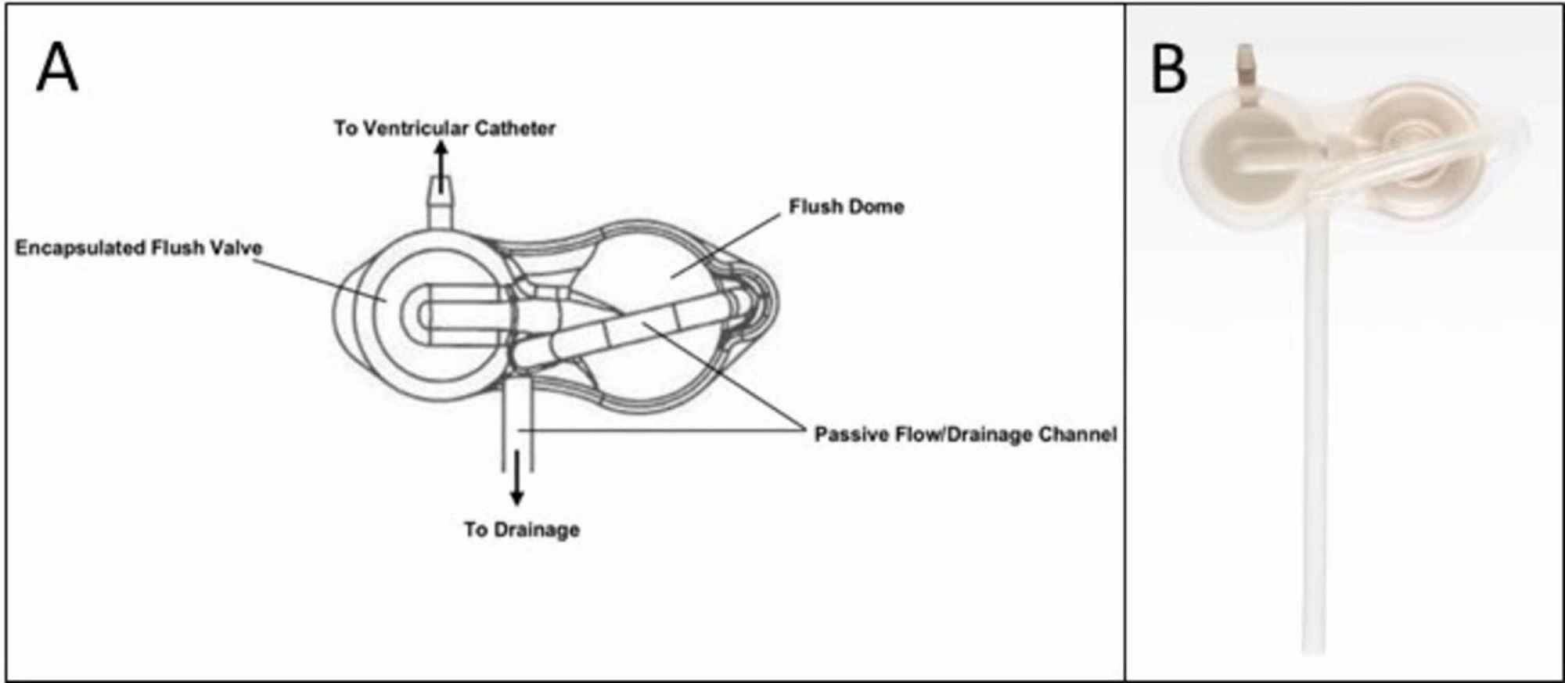
The Reflow™ flusher A. Schematic of the Reflow™ flusher and B. Image of the device A passive drainage channel passed through the flushing dome, which, when pumped, obstructs the distal flow and propels a controlled stream of CSF proximally toward the ventricles. CSF: cerebrospinal fluid

The Reflow™ flusher is a single component consisting of a flushing valve and a flushing dome. It does not itself regulate CSF flow but is designed to be connected in series with all standard shunt valves. During passive flow, fluid from the ventricular catheter flows without restriction through the passive flow channel of the flusher. During flushing, the passive channel is manually occluded to create a temporary one-way flow into the ventricular catheter. After a controlled and limited pulse of fluid is sent from the flusher and the dome is released, the flush dome refills with CSF from the ventricular catheter and resumes free-flowing through the passive flow channel (Figure 1). This introduces a means of non-invasively producing controlled, limited bursts of proximally directed flow through a catheter and out its intraventricular openings, with the potential to flush away sediment, choroid, collapsed ventricles, or other causes of obstruction.

**Figure 2:**
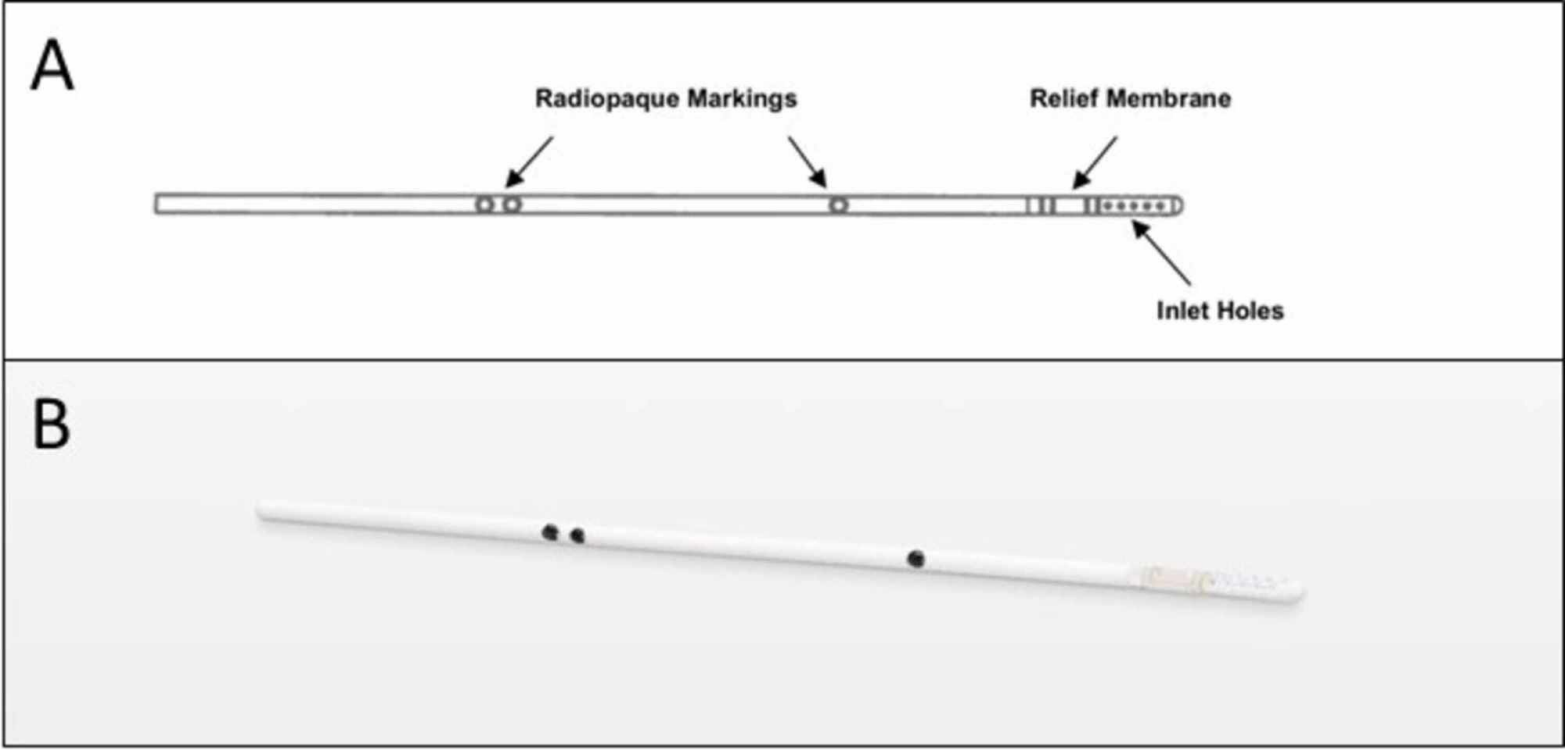
The Reflow™ ventricular catheter A. Schematic of the Reflow™ ventricular catheter and B. Image of the device. The catheter contains radiopaque markers for easy identification on imaging, as well as the relief membrane with the potential to create a new intraventricular opening when the system is flushed against obstructed inlet holes.

The second novel introduction in this system is the “relief membrane” on the proximal catheter. This is a membrane-bound side port on the catheter located just above the intraventricular inlet holes (Figure 2), which would be located within the ventricles with any standard catheter placement. During normal function and flushing of an unobstructed shunt, this membrane remains intact. When the Reflow™ flusher is pumped and proximal flow is directed against a severely occluded catheter, pressure builds and the membrane ruptures open, creating a new intraventricular opening and restoring normal anterograde flow. The pressure required to open this membrane has been measured as between 5 and 15 psi (corresponding to 258-775 mmHg), which will not occur physiologically without flushing against an occluded shunt.

## Results

Table 1 describes the baseline characteristics of the selected patients. Patient ages ranged from two to 34 years, with an average age of 11.63 (SD 8.21). Our patients were 44% female and 56% male. The underlying etiologies of hydrocephalus in these patients was variable. Ten (40%) were diagnosed with congenital hydrocephalus, all with a history of multiple prior shunt failures. Four (16%) carried a diagnosis of spina bifida. Four (26%) had a neonatal hemorrhage, two (8%) had hydrocephalus secondary to tumor, two (8%) with a spontaneous hemorrhage, one (4%) with hemorrhage secondary to arteriovenous malformation (AVM), and one (4%) with aqueductal stenosis. Follow-up times ranged from one to 22 months, with a mean of 7.18 months (SD 4.39).

**Table 1:** Patient demographics

Patient Demographics		
Age		
	Range (years)	2-34
	Average	11.63 (SD 8.21)
Sex		
	Female	11 (44%)
	Male	14 (56%)
Etiology of Hydrocephalus		
	Congenital hydrocephalus	10 (40%)
	Spina bifida	4 (16%)
	Neonatal hemorrhage	4 (16%)
	Tumor	2 (8%)
	Spontaneous hemorrhage	2 (8%
	Ruptured AVM	1 (4%)
	Aqueductal stenosis	1 (4%)

Seven individual patients presented during our observation period with symptoms concerning for shunt malfunction. Three of these improved with flushing of their reflow devices, avoiding additional invasive workups such as shunt taps or operative revisions. One was a six-year-old with spina bifida who returned with profound irritability 10 days after the placement of a new shunt with a Reflow™ system. The Reflow™ dome was pumped once by the physician and after a few hours of observation, the symptoms resolved. Another was a 16-year-old with congenital hydrocephalus with a history of frequent shunt failure who presented with symptoms of shunt failure that improved after being pumped once.

**Figure 3:**
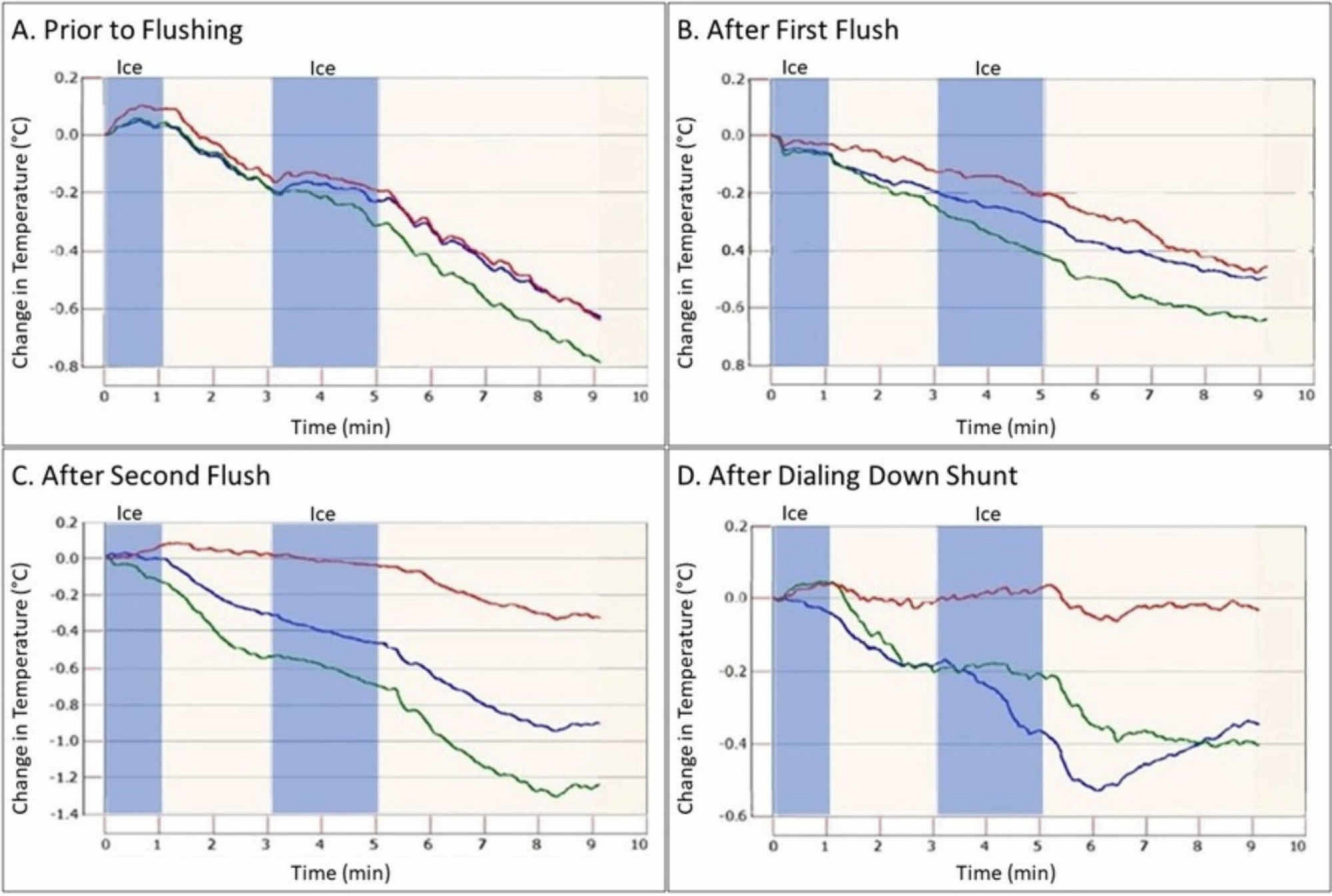
Demonstration of differential thermal dilution by means of ShuntCheck® Application of ice (blue background) over shunt tubing proximal to a sensor cools the skin at an expected rate. The flow of CSF through the shunt cools the overlying skin faster than the surrounding tissue. Figures A and B show the skin overlying the shunt (blue line) cooling at equal rates to the skin on either side (green and red lines), indicating a lack of CSF flow. Figure C demonstrates slight differential cooling, suggesting the return of CSF flow after the second flushing. Due to the suboptimal placement of sensors, both the blue and the green sensors were overlaying the shunt tubing, however, these both provide a clear contrast to the adjacent red sensor over normal skin. This differential is confirmed in D after the shunt is dialed down to maximize this effect, showing brisk flow. CSF: cerebrospinal fluid

The third was an 18-year-old with obstructive hydrocephalus treated with the Reflow™ system who presented with headache. The shunt was interrogated with the Shunt Check system. This system, based on the concept of thermal dilution to assess relative changes in temperature of skin overlying shunt tubing distal to an icepack compared to surrounding skin, uses a change in temperature of 0.2 degrees Celsius as a cut-off to confirm flow [9]. On initial evaluation, the patient was seen to have a 0.0-degree temperature drop, which persisted after one pumping by the physician. After a second pumping, a temperature drop of just over 0.1 was seen, suggesting the establishment of slow flow. The patient’s Strata shunt was then dialed from performance status 2.0 to 0.5 to maximize this effect, at which point a drop of greater than 0.2 was seen, confirming brisk flow (Figure 3). The patient’s headache gradually improved, and she was able to be discharged the next day without operative intervention.

Five operative revisions were performed in four patients during this period. One patient was seen to have debris in the proximal catheter on initial revision and was presumed to be a failure due to proximal obstruction. A second Reflow device was, therefore, placed, however, his symptoms did not improve and additional workup determined intra-abdominal adhesions causing elevated intra-abdominal pressure to be responsible. He improved after a second revision with a different terminal site. Another patient was seen to have debris in the proximal catheter and improved after operative revision, indicating a proximal obstruction that did not respond to flushing. Other causes for revision included distal tubing disconnection due to trauma and one case of surgical site infection.

Of all patients presenting with symptoms of shunt failure, three out of seven (43%) improved with flushing. Of the four patients ultimately concluded to have a proximal obstruction, either evidenced by a revision or presumed due to symptomatic improvement, three out of four (75%) improved with flushing. Of note, however, none of these cases have a definitive demonstration of the resolution of ventriculomegaly with pre and post-flushing imaging.

## Discussion

Mechanical failure of ventricular shunts has been recognized as a significant problem since their introduction in the 1950s, and despite technological advances, there has not been a significant decrease in the rate of shunt failure since that time [10]. Failure is seen at a rate of 31.3% in the first year and 4.5% per year after that [10]. The probability of shunt failure within the first decade of placement has been estimated at between 66%-81% [11-12], however, failure has been seen even 20 years beyond the initial placement [13]. Catheter occlusion is the largest cause of shunt failure [12,14-15], with proximal catheter occlusion being most common in the pediatric population [11].

The Reflow™ Ventricular System (Anuncia, Inc., Lowell, MA) is a recent development in shunt technology with the potential to prolong shunt lifespan and decrease revision rate. The Reflow™ flusher offers the unique ability to non-invasively flush a shunt proximally into the ventricle. In the setting of shunt malfunction, this introduces a mechanism to clear the proximal shunt and the inlet holes of debris, which may be contributing to obstruction. Theoretically, patients with factors associated with higher rates of shunt failure, such as trauma, infection, and hemorrhage, which cause elevated CSF protein [16], or slit-like ventricles, which may readily collapse around a catheter, could benefit from this device.

In this series, we present our experiences with the first clinical applications of this technology. We have seen that this system, which is designed to be connected in series with any commercially available shunt valve, does not impair the normal functioning of these valves. While it is possible that the added tortuosity of the flusher may theoretically increase system resistance to CSF flow, we did not see evidence that this had a clinically significant effect. Several of our patients did require shunt revision, however, in all cases, this was due to complications unrelated to the device itself. We did see one case of shunt malfunction ultimately attributable to proximal shunt obstruction, which did not respond to proximal flushing.

While we have yet to see a definitive demonstration of shunt rescue by means of flushing open inlet holes or opening of the “rescue membrane,” the three cases discussed are suggestive of the reestablishment of CSF flow through the shunt. These patients all presented with symptoms of shunt failure, which resolved after flushing of their shunts, preventing the need for additional evaluation methods or shunt revision. The demonstration of improvement in shunt flow as measured by thermodilution in one of these patients is the strongest evidence for shunt rescue at this time. The ShuntCheck® System (NeuroDx Development, Yardley, PA), which measures differential temperature changes in the skin overlying a cooled shunt catheter compared to passive heat conduction in the surrounding skin, has been shown to be a sensitive and specific measure of CSF flow [9].

There are multiple limitations that prevent definitive conclusions from this work. We have yet to see definitive proof of the flushing system and “rescue membrane” salvaging an obstructed shunt, as this would entail imaging of ventriculomegaly, which resolved after flushing. Additionally, this case series is small in sample size and poorly powered, which limits statistical analysis. The follow-up period is also quite short, and long-term outcomes remain to be evaluated. It remains unknown how long a salvaged shunt may remain viable if the primary problem is a continual buildup of debris, which may then obstruct the newly created opening. While no obstruction of the loop through the reflow flushing device was seen here, it is a theoretical possibility that this increased surface area and tortuosity of this CSF pathway may increase the risk of protein buildup and obstruction at this site, and long-term follow-up will be needed to evaluate this.

## Conclusions

After half a century of slow progress in the treatment for hydrocephalus, reduction in shunt failure remains the foremost goal of research endeavors. The newly introduced Reflow™ Ventricular System has the theoretical potential to prolong shunt lifespan and decrease the rate of shunt revision, thereby reducing patient morbidity and system-wide healthcare costs. In this initial series, we have demonstrated the use of this device in clinical practice. We present preliminary evidence of the potential of the flushing mechanism to non-invasively salvage proximally obstructed shunt catheters. Many questions remained to be answered, and long-term assessment and clinical studies of these patients will be necessary to show what potential impact this new system may have on the comprehensive management of hydrocephalus.
